# A Tissue-Specific Landscape of Alternative Polyadenylation, lncRNAs, TFs, and Gene Co-expression Networks in *Liriodendron chinense*

**DOI:** 10.3389/fpls.2021.705321

**Published:** 2021-07-23

**Authors:** Zhonghua Tu, Yufang Shen, Shaoying Wen, Huanhuan Liu, Lingmin Wei, Huogen Li

**Affiliations:** ^1^Key Laboratory of Forest Genetics & Biotechnology of Ministry of Education, Nanjing Forestry University, Nanjing, China; ^2^Co-Innovation Center for Sustainable Forestry in Southern China, Nanjing Forestry University, Nanjing, China

**Keywords:** *L. chinense*, hybrid sequencing, alternative polyadenylation, transcription factors, tissue-specific genes, gene co-expression modules, lncRNAs

## Abstract

*Liriodendron chinense* is an economically and ecologically important deciduous tree species. Although the reference genome has been revealed, alternative polyadenylation (APA), transcription factors (TFs), long non-coding RNAs (lncRNAs), and co-expression networks of tissue-specific genes remain incompletely annotated. In this study, we used the bracts, petals, sepals, stamens, pistils, leaves, and shoot apex of *L. chinense* as materials for hybrid sequencing. On the one hand, we improved the annotation of the genome. We detected 13,139 novel genes, 7,527 lncRNAs, 1,791 TFs, and 6,721 genes with APA sites. On the other hand, we found that tissue-specific genes play a significant role in maintaining tissue characteristics. In total, 2,040 tissue-specific genes were identified, among which 9.2% of tissue-specific genes were affected by APA, and 1,809 tissue-specific genes were represented in seven specific co-expression modules. We also found that bract-specific hub genes were associated plant defense, leaf-specific hub genes were involved in energy metabolism. Moreover, we also found that a stamen-specific hub TF *Lchi25777* may be involved in the determination of stamen identity, and a shoot-apex-specific hub TF *Lchi05072* may participate in maintaining meristem characteristic. Our study provides a landscape of APA, lncRNAs, TFs, and tissue-specific gene co-expression networks in *L. chinense* that will improve genome annotation, strengthen our understanding of transcriptome complexity, and drive further research into the regulatory mechanisms of tissue-specific genes.

## Introduction

*Liriodendron chinense* (*L. chinense*) is a deciduous tree species in the magnolia family (Magnoliaceae), which has desirable flowers and leaves with a unique shape. *L. chinense* is distributed throughout southern China and northern Vietnam and plays increasingly important economic and ecological roles because of its value in landscaping, furniture manufacturing and pharmaceutical manufacturing (Hao et al., 1995; Zhang et al., 2011; Xu et al., 2017). The whole-genome data of *L. chinense* have been released and contain 38 (2n = 38) chromosomes and 35,269 genes (Chen et al., 2018). However, the transcriptome of *L. chinense* has not been fully annotated, and information is lacking concerning alternative polyadenylation (APA), transcription factors (TFs), long non-coding RNAs (lncRNAs), and co-expression modules of tissue-specific genes.

Alternative polyadenylation is an important post-transcriptional regulatory mechanism that increases the complexity of the transcriptome and proteome (Sadek et al., 2019). APA causes a single gene to produce more than one transcriptome by changing the length of untranslated regions (UTRs) or coding regions (Zhang et al., 2020). APA in the UTR allows genes to produce different isoforms that have distinct UTR lengths but encode the same protein. Changes in the UTR length may affect mRNA stability, translation efficiency, or subcellular localization because UTRs usually harbor the target sites of microRNAs (miRNAs), RNA binding proteins, and other functional non-coding RNAs, and changes in the UTR length may lead to the gain or loss of these target sites (Chen et al., 2017b; Mayya and Duchaine, 2019; Zhang et al., 2020). These changes eventually affect the physiological and biochemical processes of plants. Ye et al. (2019) reported that genes with APA sites participate in cell wall formation and DNA repair under Cd stress. Cao et al. (2019) found that APA events affected gene expression and played roles in cell wall modification and root hair development. Yan et al. (2021) found that APA was involved in gene regulation in response to temperature stress. Although APA is a crucial post-transcriptional regulatory mechanism, it has not been unveiled in *L. chinense*.

lncRNAs are non-coding RNAs with lengths greater than 200 nucleotides. lncRNAs participate in transcription silencing and activation, chromosome modification, intranuclear transport, and protein modification by interacting with macromolecules (Quinn and Chang, 2016; Wu et al., 2020). Wang and Chang (2011) summarized four mechanisms of lncRNA regulation: signaling, decoying, guiding, and scaffolding. According to their location in the genome, lncRNAs can be divided into four categories: sense intronic lncRNAs, antisense lncRNAs, large intergenic lncRNAs (lincRNAs), and sense overlapping lncRNAs (Harrow et al., 2012; Wu et al., 2020). These lncRNAs participate in multiple biological processes, including plant growth and development, the stress response, disease resistance, the immune response, and vernalization response (Liu et al., 2020; Wu et al., 2020); for example, *ASCO* and *COLDAIR* participate in *Arabidopsis thaliana* root development and the vernalization response, respectively (Heo and Sung, 2011; Bardou et al., 2014), *ALEX1* enhances rice (*Oryza sativa*) disease resistance (Yu et al., 2020), and *PILNCR1* improves the tolerance of maize (*Zea mays*) under phosphate deficiency conditions (Du et al., 2018). In our previous study, we identified only 183 lncRNAs that had undergone alternative splicing (AS), and this value was far lower than the number of lncRNAs found in other species (Liu et al., 2018; Guo et al., 2020; Tu et al., 2020). Therefore, it is necessary to comprehensively study lncRNAs in *L. chinense*.

Tissue-specific genes can be regulated by APA and lncRNAs, which affect the biological traits and phenotypic characteristics of plants. Additionally, tissue-specific genes play fundamental roles in tissue differentiation and maintenance (Pattison et al., 2015). The divergence of tissue-specific gene expression patterns was found to modulate ion maintenance in tissues and improve salinity tolerance in *Populus euphratica* (Yu et al., 2017). In pineapple (*Ananas comosus*), 273 tissue-specific genes were detected, providing a basis to study the genetic mechanisms that regulate fruit phenotypes (Mao et al., 2018). Additionally, tissue-specific genes are involved in gibberellin metabolism in banana (*Musa* spp.) (Chen et al., 2016). Although studies of *L. chinense* tissues (leaves and petals) have been reported, the patterns of tissue-specific genes remain unknown, and this lack of knowledge impedes our understanding of tissue traits in this species (Yang et al., 2014; Ma et al., 2018).

Next-generation sequencing (NGS) is a high-throughput technique with high accuracy and a low cost and has been widely applied in plant transcriptome and genome research. However, it has weaknesses, such as difficulties in sequence assembly, the possibility of producing low-quality transcripts, and the inability to effectively capture full-length transcripts (Abdel-Ghany et al., 2016; Wang et al., 2016; Chen et al., 2017a; Wang et al., 2017; Chao et al., 2018, 2019; Zhang et al., 2019). Single-molecule long-read sequencing technology (SMRT) has been used in animals and plants to identify lncRNAs, comprehensively analyze APA, correct misannotated gene models and uncover novel genes (Xu et al., 2015; Abdel-Ghany et al., 2016; Chen et al., 2017a; Li et al., 2018; Liu et al., 2018; van Dijk et al., 2018; Wang et al., 2018a, b; Chao et al., 2019; Zhang et al., 2019). However, SMRT has the shortcomings of a high error rate (∼13%) and low throughput (van Dijk et al., 2018; Zhang et al., 2019). To overcome these obstacles, the use of hybrid sequencing (combining NGS with SMRT) to study the transcriptome has become an effective strategy that has been successfully applied to study various plants, such as moso bamboo (*Phyllostachys edulis*) (Zhao et al., 2018), sugarcane (*Saccharum officinarum*) (Hoang et al., 2017), and *Medicago sativa* (Chao et al., 2019). In our previous study, hybrid sequencing was applied to study AS, but these hybrid sequencing data could provide other important information in addition to AS, such as APA, TF families, the number of lncRNAs in the genome, and co-expression modules of tissue-specific genes.

Although the reference genome and AS in *L. chinense* have been reported, the annotation of APA, TFs and lncRNAs is incomplete, and the co-expression network of tissue-specific genes remains unknown. Therefore, to obtain information on APA, TFs, lncRNAs, and tissue-specific gene co-expression networks, we used hybrid sequencing data from bracts, sepals, petals, pistils, stamens, shoot apices, and leaves. Our study revealed the effects of APA on *L. chinense*, providing a landscape of lncRNAs and TFs, and uncovering the co-expression networks of tissue-specific genes. Additionally, we identified twenty hub tissue-specific genes, including two TFs, which are involved in different pathways, such as stamen development, shoot apical meristem function regulation, and plant defense. Taken together, this work will improve genome annotation and drive further research into tissue-specific functions in *L. chinense*.

## Materials and Methods

### Plant Materials

Twenty-six-year-old *L. chinense* trees located in a provenance trial plantation in Xiashu, Jurong County, Jiangsu Province (119°13′E, 32°7′N) were used as materials for sequencing. Plant samples were collected from the shoot apexes, expanded flower buds and mature leaves on April 15, 2019. The flower bud samples were divided into bracts, sepals, petals, stamens, and pistils. Each sample included three biological replicates. Twenty-one samples were frozen in liquid nitrogen and then stored at –80°C.

### SMRT and Illumina Sequencing

Total RNA isolation, library construction, sequencing, and row data processing were fully described in our previous study (Tu et al., 2020). In total, 21 Illumina sequencing libraries and 1 mixed SMRT library were constructed, sequenced, and analyzed.

### PacBio Iso-Seq Data Analysis

GMAP software was applied to align the polished consensus reads (long reads) to the reference genome (Wu and Watanabe, 2005). The polished reads were classified into five categories: (a) unmapped, (b) multiply mapped, (c) uniquely mapped, (d) mapped to ‘+’ sequences (sense sequences of the genome), and (e) mapped to ‘–’ sequences (antisense sequences of the genome). According to the mapping results, reads that were aligned to the unannotated regions of the reference genome were defined as novel genes. Seven databases (the NT, GO, Pfam, KEGG, NR, KOG, and Swiss-Prot databases) were used to annotate the functions of unmapped transcripts and novel genes (Kanehisa and Goto, 1999; Tatusov et al., 2003; Pruitt et al., 2007; Young et al., 2010; Finn et al., 2016; The UniProt, 2017).

Four additional tools (PLEK, CNCI, CPC, and the Pfam database) were used to predict lncRNAs, all with default parameters (Kong et al., 2007; Sun et al., 2013; Li et al., 2014; Finn et al., 2016). Transcripts with no potential coding sequence were retained as our set of candidate lncRNAs.

We used the TAPIS pipeline to predict APA sites (Abdel-Ghany et al., 2016) and MEME to analyze the nucleotide composition of the sequences upstream (–50 nt) and downstream (+50 nt) of all APA sites for nucleotide bias (Alamancos et al., 2015).

TFs were identified using iTAK software with the default parameters (Zheng et al., 2016), and expression trend analysis was performed using the OmicStudio tools (STEM package) at https://www.omicstudio.cn/tool.

### Identification of Differentially Expressed Genes and Tissue-Specific Genes and GO/KEGG Enrichment Analyses

The number of reads that mapped to each gene was calculated by Cuffdiff (Sreya and Chon-Kit Kenneth, 2016). The fragments per kilobase of transcript sequence per million mapped reads (FPKM) value of each gene was calculated based on the read count mapped to the gene and length of the gene.

DEseq2 software^1^ was used for differential expression analysis. Genes with adjusted *p*-values that were less than 0.05 and | log2fold change| ≥ 1 were defined as differentially expressed genes (DEGs). Based on the differential expression analysis results, tissue-specific genes were determined according to the following conditions: the FPKM value must be greater than 1 in a specific tissue, and the expression level of tissue-specific genes must be at least fourfold higher than that in other tissues (Mortazavi et al., 2008; Wang et al., 2019). GO enrichment analysis was then performed using GOseq software^2^. KOBAS software^3^ was used to perform KEGG pathway enrichment analysis.

### Gene Co-expression Network Analysis and Identification of Hub Genes

Gene co-expression analysis was performed via weighted correlation network analysis (WGCNA) in R software (Langfelder and Horvath, 2008). Of the 21,944 DEGs between the seven tissues, we selected genes that ranked in the top 75% according to the ranking of the FPKM variance values. The most suitable power was then determined (14 in this study) according to the calculation results of the soft threshold power using the pickSoftThreshold function (the soft threshold power ranged from 1 to 30). Next, the blockwiseModules function was applied to construct gene co-expression networks using the following parameters: a power of 14, an unsigned TOM type, a min module size of 200, a mergeCutHeight of 0.2, and default values of the other parameters. Based on the WGCNA results, we filtered edges with weight values less than 0.3. The degree, MCC, EPC, closeness, and radiality algorithms of the CytoHubba package in Cytoscape software^4^ were then used to select hub genes; genes that ranked in the top 2% by all algorithms were identified as hub genes (Smoot et al., 2011). The gene co-expression networks were visualized using Gephi software^5^.

### Validation of Hybrid Sequencing

RT-qPCR was used to validate the expression levels of five tissue-specific genes and 16 DEGs. In each sample, 1 μg of total RNA was used to synthesize cDNA using PrimeScript^TM^ RT Master Mix (TaKaRa, Dalian, China). The Oligo 7 algorithm^6^ was used to design RT-qPCR primers for the 16 selected DEGs (Supplementary Table 1). RT-qPCR was performed using a StepOnePlus^TM^ system (Applied Biosystems) with SYBR^®^ Premix Ex Taq^TM^ (TaKaRa, Dalian, China), as directed by the instructions. *Eukaryotic translation initiation factor 3* (*eIF3*) was designated as an internal control gene (Tu et al., 2019).

## Results

### Processing of the PacBio Sequencing Data

Using PacBio SMRT, we obtained 618,876 polymerase reads from 21 pooled samples (Supplementary Table 2). From the polymerase reads, 10,437,029 subreads were extracted, with an average length of 2,177 bp. After further processing, 498,059 circular consensus sequences (CCSs) were obtained, each containing a poly(A) tail and 5′ and 3′ adaptors, with an average length of 2,755 bp. Among the CCSs, we detected 430,554 full-length non-chimeric (FLNC) reads, with an average length of 2,568 bp. The reads were polished with both iterative clustering for error correction (ICE), resulting in 227,276 polished consensus reads whose average length was 2,697 bp. By mapping the short reads generated from Illumina sequencing via LoRDEC, we corrected up to 100% of the sequencing errors (Salmela and Rivals, 2014). We ultimately obtained 227,276 high-quality polished consensus reads with an average length of 2,696 bp for further analysis (Supplementary Table 3).

**FIGURE 1:**
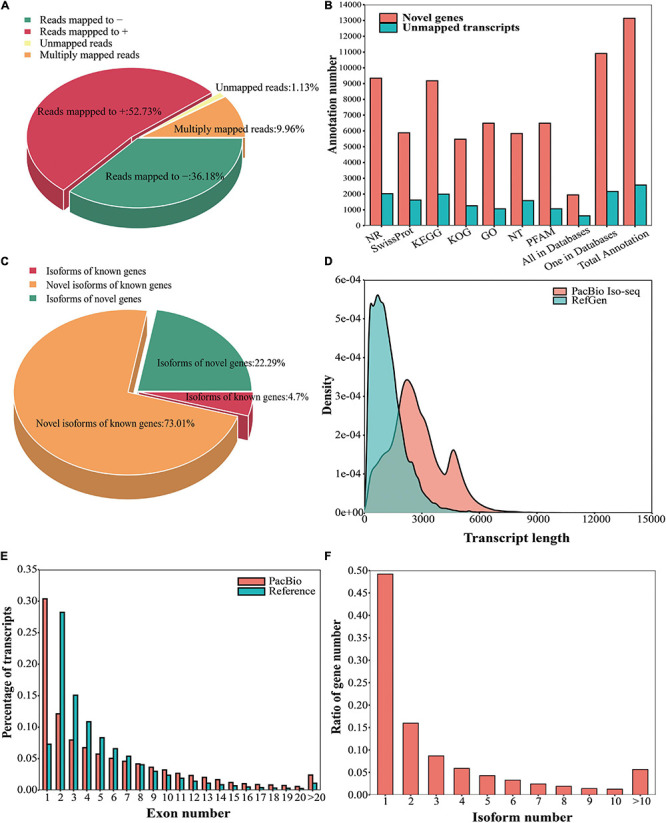
Analysis of the Iso-Seq isoforms. **(A)** GMAP mapping results (‘+’ represents the sense sequence of the genome, and ‘–’ represents the antisense sequence of the genome). **(B)** Functional annotation of the unmapped transcripts and novel genes in seven databases. **(C)** Classification of transcripts based on the reference genome. **(D)** Comparison of the isoform lengths between the PacBio data and reference genome annotation. **(E)** Comparison of the number of exons in the transcripts between the PacBio data and reference genome annotation. **(F)** Number of transcripts produced by each gene according to PacBio Iso-Seq.

GMAP software was used to align the high-quality polished consensus reads to the reference genome, and 1.13% (2,572/227,276) of the reads were found to be unmapped (Wu and Watanabe, 2005). Among the remaining 224,704 alignments, 9.96% (22,628/227,276) were mapped to multiple positions, and 52.73% (119,840/227,276) and 36.18% (82,236/227,276) were mapped to the sense sequences and antisense sequences in the genome, respectively (Figure 1A). After removing consensus reads with incomplete 5′ and 3′ ends and reads from fusion genes, 144,006 complete consensus reads could be mapped to the reference gene model (Supplementary Table 4).

In the PacBio sequencing data set, 2,572 unique clusters did not align to the reference genome. These unmapped transcripts were then mapped to 7 databases: NR, NT, Pfam, KOG, Swiss-Prot, KEGG, and GO. In these databases, 2,022 (NR), 1,584 (NT), 1,066 (Pfam), 1,260 (KOG), 1,621 (Swiss-Prot), 1,995 (KEGG), and 1,066 (GO) unmapped transcripts were annotated (Figure 1B). Among these unmapped transcripts, 619 were found in all seven databases, and 2,163 were annotated in only one database (Figure 1B).

We defined reads that mapped to unannotated regions of the reference genome as reads from novel genes, and 13,139 novel genes covered by 31,792 complete consensus reads were detected (Supplementary Table 3). To better understand the novel genes, they were functionally annotated. In total, 9,343 (NR), 5,833 (NT), 6,494 (Pfam), 5,478 (KOG), 5,883 (Swiss-Prot), 9,177 (KEGG), and 6,494 (GO) novel genes were annotated in the seven databases (Figure 1B). Additionally, 1,945 novel genes were annotated across all seven databases, and 10,905 novel genes were annotated in only one database (Figure 1B).

### Isoform Detection and Characterization

TAPIS software was used to classify and characterize the full-length isoforms (Abdel-Ghany et al., 2016). A total of 14,488 known genes covered by 69,658 isoforms, including 4,210 (4.70%) isoforms of known genes, 65,448 (73.01%) novel isoforms of known genes, and 13,139 novel genes covered by 19,982 (22.29%) isoforms (Figure 1C). We compared the lengths of the transcripts between the PacBio data and reference genome annotation and found that the transcripts described in the reference genome annotation were shorter than those detected by PacBio Iso-Seq (Figure 1D). Additionally, we compared the numbers of exons in the transcripts between the PacBio data and reference genome annotation. Single-exon genes represented a large proportion (30.37%) of the PacBio data; however, in the reference genome, single-exon genes accounted for only 7.29% (Figure 1E). The proportion of multiple-exon genes (≥2 exons) in the reference genome annotation was greater than that in the PacBio data (Figure 1E). Furthermore, single-isoform genes constituted a large proportion of the PacBio data (49.22%), followed by two-isoform genes, which accounted for 15.98% (Figure 1F).

### Comparison of lncRNAs and mRNAs

**FIGURE 2:**
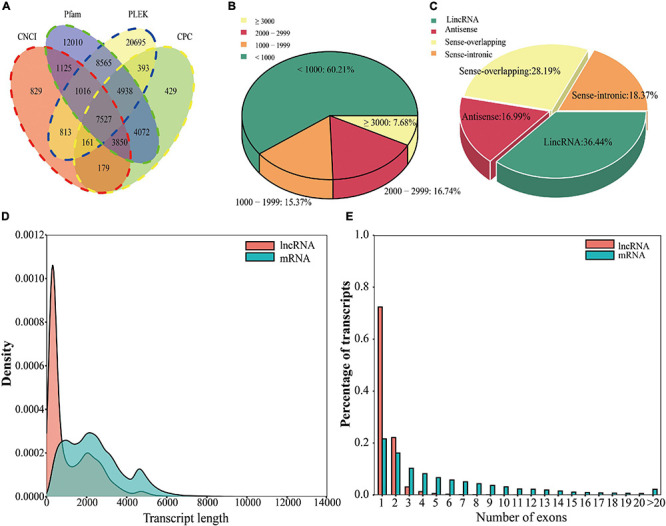
Identification, classification, and analysis of lncRNAs in *L. chinense*. **(A)** Venn diagram of lncRNAs predicted by the CNCI, Pfam, PLEK, and CPC tools. **(B)** Percentage of lncRNAs with different length. **(C)** Classification of lncRNAs. **(D)** Comparison of the lengths of mRNAs and lncRNAs. **(E)** Comparison of the numbers of exons of mRNAs and lncRNAs.

lncRNAs are RNA molecules that are longer than 200 nt that do not encode proteins. We used four tools, PLEK, CNCI, CPC, and Pfam, to identify lncRNAs in the PacBio Iso-Seq data (Kong et al., 2007; Sun et al., 2013; Li et al., 2014; Finn et al., 2016). The PLEK, CNCI, CPC, and Pfam tools predicted 44,108 lncRNAs, 15,500 lncRNAs, 21,549 lncRNAs, and 43,103 lncRNAs, respectively (Figure 2A). To improve the accuracy of the lncRNA prediction, each lncRNA was predicted by four tools, and 7,527 lncRNAs were detected by all four tools (Figure 2A). Most of the lncRNAs were less than 1,000 bp (Figure 2B). Among these, lincRNAs accounted for the largest proportion (36.44%), followed by sense overlapping lncRNAs (28.19%), sense intronic lncRNAs (18.37%), and antisense lncRNAs (16.99%) (Figure 2C; Harrow et al., 2012). Compared with mRNAs, lncRNAs generally had fewer exons and were shorter, and single-exon lncRNAs accounted for most of the lncRNAs (72.34%) (Figures 2D,E).

### Identification of DEGs and Tissue-Specific Genes

**FIGURE 3:**
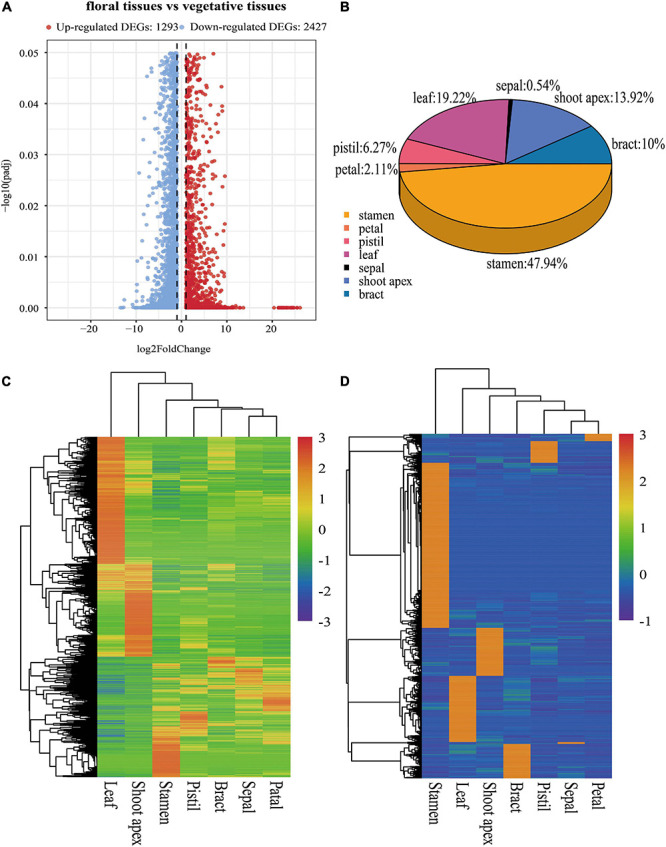
Information about DEGs and tissue-specific genes. **(A)** Volcano plot of DEGs between floral tissues and vegetative tissues. **(B)** Distribution of tissue-specific genes in seven tissues. **(C)** Hierarchical clustering analysis of DEGs based on the normalized log_10_(FPKM) values. **(D)** Hierarchical clustering analysis of tissue-specific genes.

To investigate gene expression patterns in the seven evaluated tissues of *L. chinense*, we used FPKM values to normalize the reads from Illumina sequencing. We identified 3,720 DEGs between floral tissues (bracts, sepals, petals, stamens, and pistils) and vegetative tissues (leaves and the shoot apex); 1,293 of these DEGs were upregulated, and the other 2,427 were downregulated (Figure 3A). The Euclidean distance method was used to perform clustering analysis of all the genes to identify their clustering patterns in the different tissues (Yang et al., 2018). Hierarchical clustering analysis showed that these DEGs were clustered into three subclusters, one of which was highly expressed in leaves, one in the shoot apex and the other in floral tissues (Figure 3C).

To better understand tissue-specific genes in *L. chinense*, we investigated their distribution and pathways with which these genes were associated. Overall, 2,040 tissue-specific genes in seven tissues were detected. Stamen tissue contained the highest number of tissue-specific genes (978, 47.94%), followed by the leaf (392, 19.22%), shoot apex (284, 13.92%), bract (204, 10.00%), pistil (128, 6.27%), and petal (43, 2.11%) tissues, while the sepal tissue contained the least number of tissue-specific genes (11, 0.54%) (Figure 3B). Hierarchical clustering analysis showed that these tissue-specific genes were obviously clustered into seven subclusters (Figure 3D). GO enrichment analysis results showed that 29 GO terms were significantly enriched, and the largest number of tissue-specific genes were significantly enriched in catalytic activity, followed by transferase activity and hydrolase activity (Supplementary Figure 1A). KEGG pathway enrichment analysis revealed that these tissue-specific genes were significantly enriched in nine pathways, and the largest number of tissue-specific genes were significantly enriched in the biosynthesis of secondary metabolites pathway (Supplementary Figure 1B).

### Identification and Analysis of TFs in *L. chinense*

**FIGURE 4:**
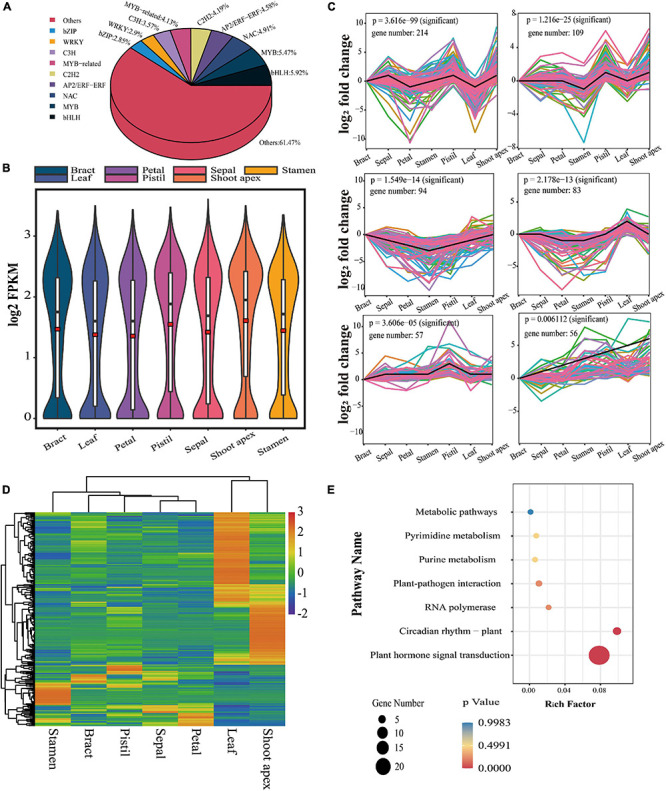
Analysis of TFs in *L. chinense*. **(A)** Proportion of different TF families (only the top 9 TF families are shown). **(B)** Expression trend analysis of TFs. **(C)** Expression levels of TFs in seven tissues; the red rectangles represent the average values. **(D)** Hierarchical clustering analysis of differentially expressed TFs. **(E)** KEGG pathway enrichment analysis of differentially expressed TFs.

We identified 1,791 TFs from 94 families, among which the bHLH family had the largest number of TFs (106, 5.92%), followed by MYB (98, 5.47%), and NAC (88, 4.91%) (Figure 4A). These 1,791 TFs showed the highest expression level in the shoot apex and the lowest expression level in petals (Figure 4B). Expression trend analysis identified only six significant expression patterns, including 613 TFs (Figure 4C). Moreover, we found that 244 TFs were differentially expressed between floral tissues and vegetative tissues, and hierarchical clustering analysis showed that these 244 TFs could be clustered into two subclusters (vegetative tissue subcluster and floral tissue subcluster) (Figure 4D). These differentially expressed TFs were significantly enriched in plant hormone signal transduction and circadian rhythm pathways (Figure 4E).

### Polyadenylation Analysis and Identification of Alternatively Spliced Tissue-Specific Genes

**FIGURE 5:**
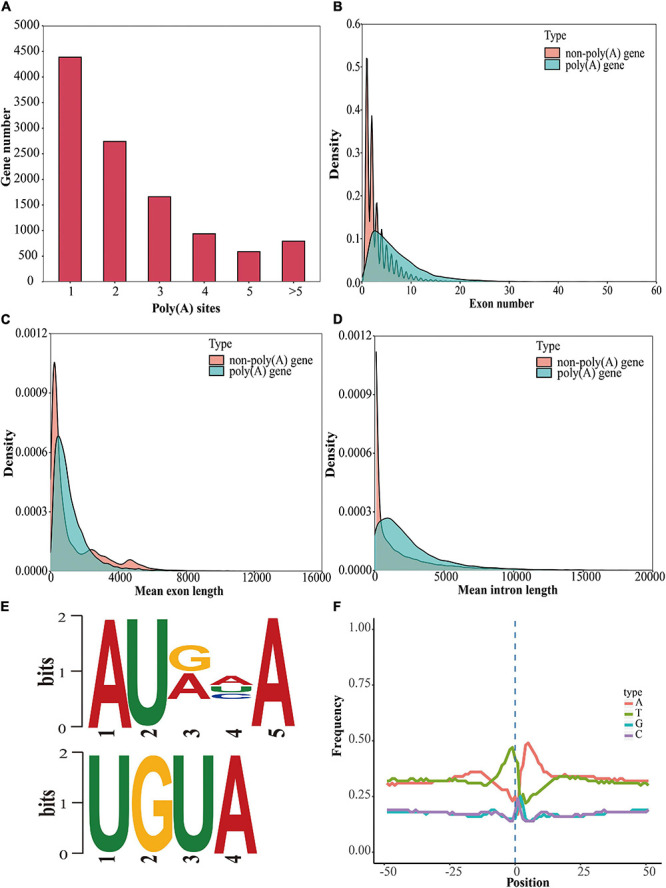
Identification and analysis of genes with poly(A) sites. **(A)** Analysis of the gene numbers according to the number of poly(A) sites. **(B)** Comparison of the exon numbers of poly(A) genes and non-poly(A) genes. **(C)** Comparison of the mean exon lengths of poly(A) genes and non-poly(A) genes. **(D)** Comparison of the mean intron lengths of poly(A) genes and non-poly(A) genes. **(E)** Motif analysis around poly(A) cleavage sites. **(F)** Nucleotide distribution around poly(A) cleavage sites.

**FIGURE 6:**
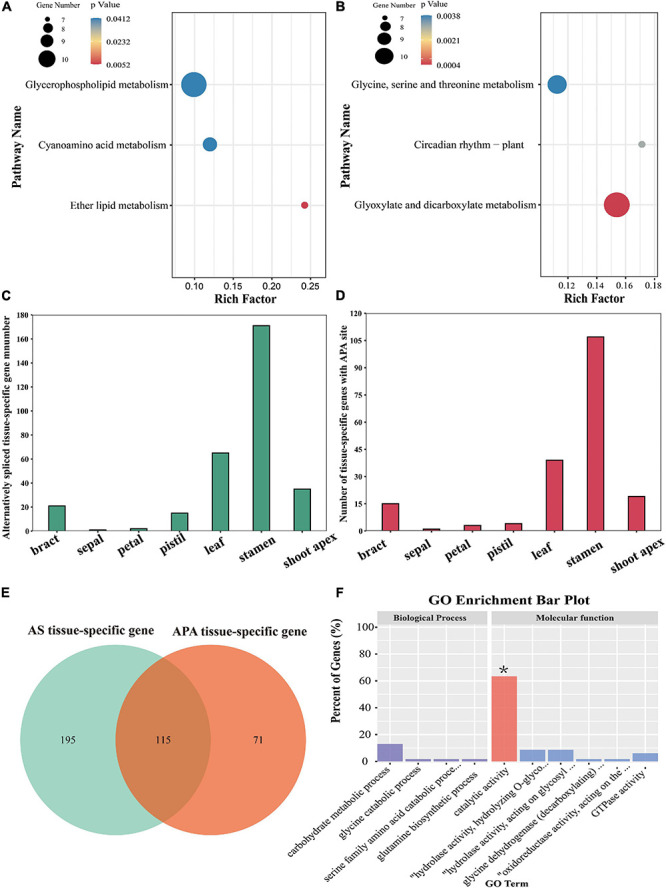
KEGG pathway enrichment analysis of the DEGs with APA sites and analysis of the tissue-specific genes affected by AS or APA. **(A,B)** KEGG pathway enrichment analysis of up-regulated and down-regulated genes affected by APA, respectively. **(C,D)** Numbers of tissue-specific genes affected by AS and APA, respectively. **(E)** Venn plot of AS tissue-specific genes and APA tissue-specific genes. **(F)** GO enrichment analyses of 115 genes affected by both AS and APA.

Most genes in eukaryotes can generate various mRNA 3′ ends through APA, markedly increasing the complexity of the transcriptome (Shen et al., 2011; Wu et al., 2011). Using the TAPIS pipeline (Abdel-Ghany et al., 2016), we identified 11,108 annotated genes containing at least one poly(A) site [poly(A) gene], among which 4,387 annotated genes comprised a single poly(A) site and 6,721 annotated genes contained more than one poly(A) site (Figure 5A). Poly(A) genes showed more exons, shorter mean exon lengths, and longer mean intron lengths than genes without poly(A) sites [non-poly(A) genes] (Figures 5B–D). To identify the potential *cis*-elements involved in polyadenylation, motif enrichment analysis was performed to analyze the 50 nucleotides upstream from the poly(A) sites (Abdel-Ghany et al., 2016). We discovered two conserved motifs (AUAAA and UGUA) upstream of the poly(A) cleavage site (Figure 5E); these motifs were also present in maize, red clover (*Trifolium pratense* L.), sorghum (*Sorghum bicolor*), *Brassica napus*, and poplar (*Populus alba* var. *pyramidalis*) (Abdel-Ghany et al., 2016; Wang et al., 2016; Chao et al., 2018; Hu et al., 2020; Yao et al., 2020). Additionally, to investigate the preferential nucleotides at the poly(A) cleavage sites, we analyzed the nucleotide composition of 50 downstream and 50 upstream nucleotides at all APA cleavage sites. Uracil (U) was found to be enriched upstream of the APA cleavage sites, while adenine (A) was enriched downstream of the cleavage sites (Figure 5F). The same findings were reported in red clover and sorghum (Abdel-Ghany et al., 2016; Chao et al., 2018). Additionally, we found that 990 APA genes were differentially expressed between floral tissues and vegetative tissues, among which 394 upregulated APA genes were significantly enriched in glycerophospholipid metabolism, cyanoamino acid metabolism, and ether lipid metabolism pathways, and 596 downregulated APA genes were significantly enriched in glycine, serine and threonine metabolism, circadian rhythm, and glyoxylate and dicarboxylate metabolism pathways (Figures 6A,B).

In our previous study, we identified 8,503 AS genes (Tu et al., 2020). Actually, 310 tissue-specific genes had undergone AS; stamen-specific genes accounted for most of these (171), followed by leaf-specific genes (65), shoot-apex-specific genes (35), bract-specific genes (21), pistil-specific genes (15), petal-specific genes (2), and a sepal-specific gene (1) (Figure 6C). Moreover, 187 tissue-specific genes were identified to have APA sites, including 107 stamen-specific genes, 39 leaf-specific genes, 18 shoot-apex-specific genes, 15 bract-specific genes, 4 pistil-specific genes, 3 petal-specific genes, and 1 sepal-specific gene (Figure 6D). We found that 115 tissue-specific genes were affected by both AS and APA, among which 73 genes were significantly enriched in catalytic activity GO (Figures 6E,F).

### WGCNA Analyses of DEGs and Tissue-Specific Genes

**FIGURE 7:**
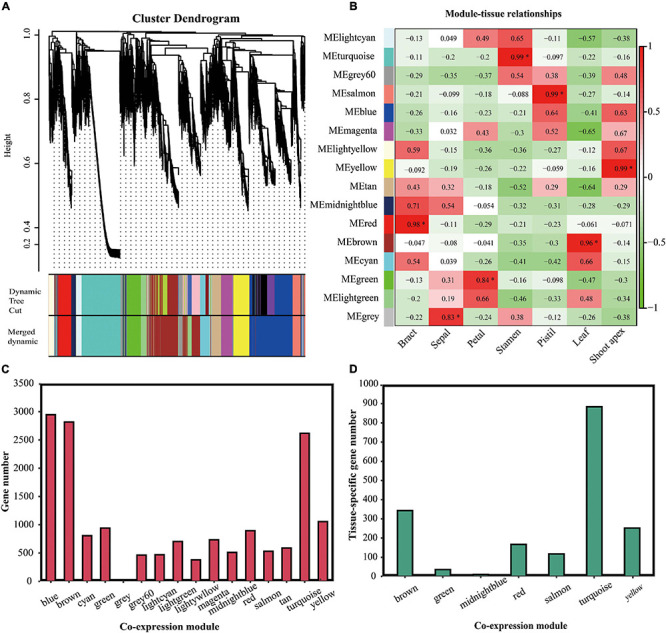
Analysis of the gene co-expression modules. **(A)** Dendrogram of the gene co-expression modules in *L. chinense*. The genes are represented by vertical lines in the upper panel. In the bottom panel, rectangles of thirteen colors represent the 16 gene co-expression modules. **(B)** Relationships between tissues and gene co-expression modules. The asterisk indicates that the co-expression module is significantly correlated to the tissue. **(C)** Analysis of the gene numbers in the 16 co-expression modules. **(D)** Analysis of the tissue-specific genes in the 16 co-expression modules.

Weighted correlation network analysis was used to analyze gene co-expression (Langfelder and Horvath, 2008). In total, 21,944 DEGs between seven tissues were identified, and only the DEGs ranked in the top 75% based on the ranking of the variance of FPKM values could be used for WGCNA. First, we identified 22 gene co-expression modules (genes that could not be clustered because of their large difference from other genes were divided into a gray module) (Figure 7A). However, some modules were highly correlated. Thus, we merged the modules with correlations higher than 0.8 (mergeCutHeight = 0.2). Finally, 16 co-expression modules were identified. The number of genes in each co-expression module ranged from 3 (gray module) to 2,946 (blue module) (Figure 7C). In total, 1,809 tissue-specific genes were classified into seven co-expression modules as follows: the brown module contained 343 leaf-specific genes, the green module contained 36 petal-specific genes, the midnight-blue module contained 10 sepal-specific genes, the red module contained 167 bract-specific genes, the salmon module contained 117 pistil-specific genes, the turquoise module contained 884 stamen-specific genes, and the yellow module contained 252 shoot-apex-specific genes (Figure 7D).

Because tissue-specific genes tended to appear in specific modules, we investigated whether there were specific co-expression modules that were strongly related to specific tissues. We analyzed the correlation between the 16 co-expression modules and 7 tissues. We found that the red, green, turquoise, salmon, brown, and yellow modules were significantly correlated with bract, petal, stamen, pistil, leaf, and shoot apex tissue, respectively (*P* < 0.05) (Figure 7B). Although other modules had relationships with certain tissues, these correlations were not significant.

Hierarchical clustering analysis showed that these 1,809 tissue-specific genes represented obvious specificity (Supplementary Figure 2). Next, we performed KEGG pathway enrichment analyses for these 1,809 tissue-specific genes in specific co-expression modules. Tissue-specific genes in specific modules were significantly enriched in the biosynthesis of secondary metabolite pathways, except for bract-specific genes in the red module and petal-specific genes in the green module (Supplementary Figure 3). Shoot-apex-specific genes, leaf-specific genes, pistil-specific genes, and stamen-specific genes were also significantly enriched in the phenylpropanoid biosynthesis pathway (Supplementary Figures 3D–G).

### Identification of Hub Genes in Specific Co-expression Modules

**FIGURE 8:**
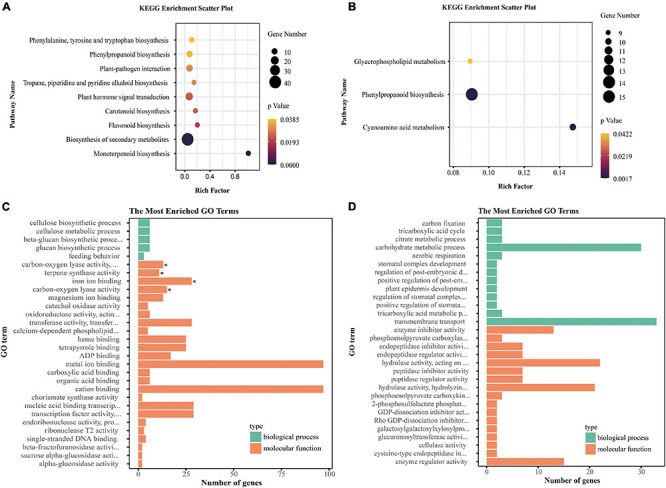
KEGG and GO enrichment analysis of genes co-expressed with two hub TFs. **(A,B)** KEGG pathway enrichment analysis of genes co-expressed with *Lchi05072* and *Lchi25777*, respectively (only significantly enriched pathways are shown). **(C,D)** GO enrichment analysis of genes co-expressed with *Lchi05072* and *Lchi25777*, respectively (only the top 30 terms according to *P*-values are shown). The asterisks represent significantly enriched terms.

As mentioned above, most of the tissue-specific genes were specifically expressed in seven co-expression modules: red module (bract-specific genes), brown module (leaf-specific genes), green module (petal-specific genes), salmon module (pistil-specific genes), midnight blue module (sepal-specific genes), yellow module (shoot-apex-specific genes), and turquoise module (stamen-specific genes). Therefore, we analyzed the co-expression networks of these tissue-specific genes in these specific modules and identified hub genes in the co-expression networks (Supplementary Figure 4). We identified 20 hub genes from seven specific modules that included two TFs, *Lchi05072* [*AINTEGUMENTA-LIKE 5* (*AIL5*), shoot-apex-specific] and *Lchi25777* (*SPL/NZZ*, stamen-specific) (Supplementary Table 5). *Lchi05072* was co-expressed with 672 genes, and *Lchi25777* was co-expressed with 578 genes (Supplementary Figure 4). We performed KEGG and GO enrichment analyses for them. Genes co-expressed with *Lchi05072* were significantly enriched in nine pathways, including the biosynthesis of secondary metabolites pathway and plant hormone signal transduction pathway, and four GO terms, including terpene synthase activity (Figures 8A,C). Genes co-expressed with *Lchi25777* were significantly enriched in three pathways, one of which was phenylpropanoid biosynthesis; however, they were not significantly enriched in any GO terms (Figures 8B,D). Additionally, we found that most of the bract-specific hub genes were associated with plant resistance, such as *Lchi00262* (*pathogenesis-related protein*, *PR*), *Lchi04173* (*L-type lectin receptor-like kinase*, *LecRLK*), and *Lchi05264* (*leucine-rich repeat receptor-like protein kinase*, *LRR-RLK*). Additionally, we found two leaf-specific hub genes, *Lchi03373* (*glyceraldehyde-3-phosphate dehydrogenase*, *GAPDH*) and *Lchi05497* (*ribulose bisphosphate carboxylase/oxygenase*, *Rubisco*), which were involved in energy metabolism (Supplementary Table 5). These hub tissue-specific genes have important research significance.

### RT-qPCR Validation for RNA Sequencing Data

**FIGURE 9:**
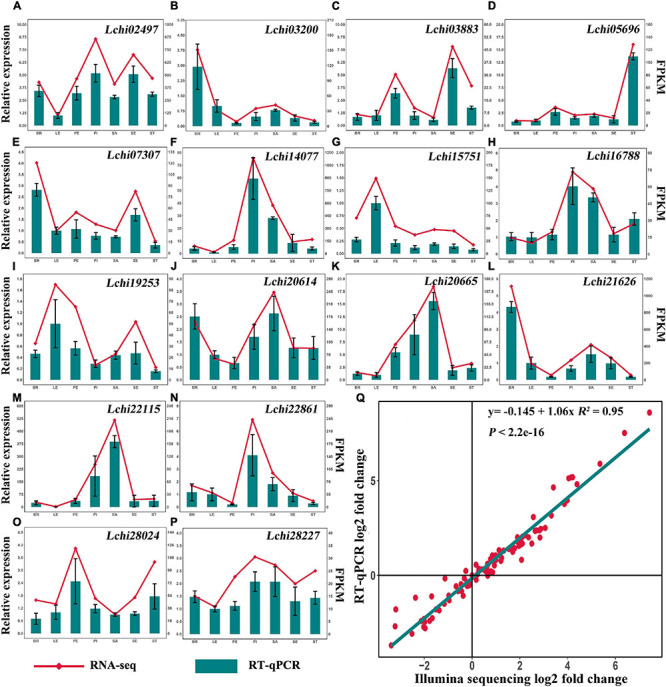
RT-qPCR validation of RNA sequencing. BR, bract; LE, leaf; PE, petal; PI, pistil; SA, shoot apex; SE, sepal; ST, stamen. **(A–P)** Relative expression analysis and FPKM values of 16 DEGs in different tissues. **(Q)** Correlation analysis of RT-qPCR data and RNA sequencing data.

To validate the accuracy of the gene expression levels detected by RNA sequencing, we performed RT-qPCR of 16 randomly selected DEGs. The expression levels of the 16 DEGs measured by Illumina sequencing were consistent with the RT-qPCR analysis results (Figures 9A–P). Correlation analysis revealed that the RNA sequencing data were highly correlated with the RT-qPCR analysis results (*R*^2^ = 0.947; *P* < 0.01) (Figure 9Q). These results indicate that the gene expression levels detected by Illumina sequencing are reliable.

## Discussion

*Liriodendron chinense*, an economically and ecologically important tree species, has received consistent research attention. Since the genome of *L. chinense* was released, the understanding of its evolutionary history has improved (Chen et al., 2018). However, the reference genome has not been fully annotated, and critical information, such as AS, APA, TFs, and lncRNAs, remains to be uncovered. In our previous study, we used hybrid sequencing to reveal the effect of AS in *L. chinense*, but this approach did not fully exploit these data to obtain more useful information, such as APA, lncRNAs, TFs, and tissue-specific genes. Thus, we conducted in-depth research and analysis of the *L. chinense* full-length transcriptome from different perspectives.

lncRNAs are essential regulators involved in plant growth and development, the stress response, disease resistance, the immune response, and the vernalization response (Wu et al., 2020). In our previous study, we identified only 183 lncRNAs affected by AS (Tu et al., 2020). However, in other studies, the number of lncRNAs was much more than 183. For example, Liu et al. (2018, 2019) detected 5,952 and 13,099 lncRNAs in poplar, Guo et al. (2020) identified 4,460 lncRNAs, and Tian et al. (2016) found 7,655 lncRNAs; thus, we speculated that 183 AS lncRNAs represented only a proportion of lncRNAs in *L. chinense*. Through in-depth analysis, we detected 7,527 lncRNAs, much more than the 183 AS lncRNAs. Compared with protein-coding genes, the effect of AS in lncRNAs was much weaker (Tu et al., 2020). Among these lncRNAs, lincRNA accounted for the largest proportion, a finding that was consistent with reports in poplar and hickory (*Carya cathayensis*) (Liu et al., 2018; Fan et al., 2020; Guo et al., 2020). Studies have revealed that lincRNAs are involved in transcriptional regulation and signal transduction in soybean (*Glycine max*) and regulate root development in *A. thaliana* (Li et al., 2016; Golicz et al., 2018). These *L. chinense* lincRNAs have potential research significance. Additionally, we found that the lncRNAs were shorter and smaller than the mRNAs in terms of the exon length and number. This phenomenon was also observed in hickory, rice, and poplar (Liu et al., 2018; Fan et al., 2020; Zhao et al., 2020). These findings provide a genome-wide landscape of lncRNAs in *L. chinense*.

Alternative polyadenylation is a critical post-transcriptional regulatory mechanism involved in maintaining RNA stability, ensuring accurate RNA localization and translation; these stabilities are crucial in plant development and flowering (Liu et al., 2010; Shen et al., 2011; Zhang et al., 2018). In *A. thaliana*, approximately 60% of the genes have multiple poly(A) sites (Shen et al., 2011). In *L. chinense*, 13.8% (6,721/48,408) of the genes had APA sites. The number and source of sequencing samples, sequencing depth, and analysis methods strongly affect the APA analysis results; thus, the proportion of detected APA genes in *L. chinense* is lower than that in other studies, likely because of the above reasons (Shen et al., 2011; Wu et al., 2011; Ruan et al., 2018). However, 26.6% of DEGs were affected by APA, indicating that APA may participate in gene expression regulation. Additionally, we found that the distribution of nucleotides upstream and downstream of the APA cleavage sites was consistent with that in previous reports of poly(A) analyses in sorghum and red clover (Abdel-Ghany et al., 2016; Chao et al., 2018). Furthermore, two motifs (AUAAA and UGUA) upstream of the poly(A) cleavage sites were detected. AUAAA is a canonical *cis* element that plays a role as a polyadenylation signal (PAS) for 3′ end processing and has been reported in red clover, sorghum and other plant species (Abdel-Ghany et al., 2016; Wang et al., 2016; Chao et al., 2018; Turner et al., 2018). The UGUA motif is a U-rich element (USE) that cooperates with auxiliary elements to affect 3′ end processing (Turner et al., 2018). In the absence of PAS, the cleavage factors bind to the 3′ end processing machinery with the help of USE or auxiliary elements (Venkataraman et al., 2005). These findings demonstrate that APA contributes substantially to the complexity and flexibility of the *L. chinense* transcriptome.

As a special category of DEGs, tissue-specific genes play important roles in plant defense, stress responses, plant development and material metabolism (Pattison et al., 2015; Celedon et al., 2017; Villarino et al., 2017; Yu et al., 2017; Alonso-Serra et al., 2019; Amini et al., 2019). In *Ferula asafetida*, genes involved in terpenoid and phenylpropanoid metabolism are expressed in flowers (Amini et al., 2019). In tomato (*Solanum pennellii*), tissue-specific genes play important regulatory roles during fruit development (Pattison et al., 2015). In the present study, we identified 2,040 tissue-specific genes in *L. chinense*. Enrichment analysis revealed that tissue-specific genes participated in multiple metabolism and synthesis pathways, such as starch and sucrose metabolism, phenylpropanoid biosynthesis, and flavonoid biosynthesis. Previous studies have found that stamen development is accompanied by starch accumulation and degradation, and phenylpropanoid biosynthesis is essential for pollen development (Julian et al., 2011; Xue et al., 2020). Flavonoids are important products of secondary metabolism and participate in multiple functions in plants, such as developmental regulation, flower pigmentation, and stress responses (Mathesius, 2018). Moreover, most of the tissue-specific genes co-expressed in specific modules, providing a landscape of co-expression networks of tissue-specific genes in *L. chinense*.

Analysis of the co-expression network of tissue-specific genes in specific co-expression modules identified two tissue-specific TFs (*AIL5* and *SPL/NZZ*) with important research value that were also hub genes in the co-expression modules. A previous study revealed that *AIL* genes synergistically regulate the function of the shoot apical meristem (Mudunkothge and Krizek, 2012). However, other studies showed that *AIL5* contributes to flower development (Nole-Wilson et al., 2005; Krizek, 2015). As a shoot-apex-specific hub gene, the function of *LcAIL5* need be further studied. Additionally, studies revealed that *SPL/NZZ* participates in stamen identity determination and is essential for anther development in *A. thaliana* (Liu et al., 2009; Zhao et al., 2017; Li et al., 2019). *SPL/NZZ* was reported to be expressed in stamen primordia and developing stamens (Zhao et al., 2017); in our study, *SPL/NZZ* was specifically expressed in the stamen. Additionally, genes co-expressed with *SPL/NZZ* were significantly enriched in phenylpropanoid biosynthesis. Phenylpropanoid derivatives are important components of sporopollenin, and the pollen exine comprises sporopollenin, which protects the pollen spore (Xue et al., 2020). Thus, we speculate that *LcSPL/NZZ* may be involved in stamen identity determination and anther development regulation, similar to *AtSPL/NZZ*, but this hypothesis required further verification. Furthermore, we found that bracts may be an important component of the *L. chinense* defense system to protect other floral tissues because most of the bract-specific hub genes were related to plant defense, such as *PR*, *LecRLK*, and *LRR-RLK*. Previous studies have shown that *PR*, *LecRLK*, and *LRR-RLK* play roles in plant immunity and pathogen defense (Parrott et al., 2016; Wang and Bouwmeester, 2017; Farrakh et al., 2018). These findings indicate that tissue-specific genes play a significant role in maintaining tissue characteristics in *L. chinense.*

Overall, this work enhanced our knowledge of the effect of APA on *L. chinense* transcriptome complexity and provided a landscape of lncRNAs and tissue-specific gene co-expression modules. This study also identified two hub TFs and 18 tissue-specific hub genes with important research value that were essential for maintaining tissue characteristics.

## Data Availability Statement

The datasets presented in this study can be found in online repositories. The names of the repository/repositories and accession number(s) can be found in the article/Supplementary Material.

## Author Contributions

ZT and HL: experimental design. ZT, YS, HL, and SW: plant material collection and performing the experiments. ZT, YS, and LW: data analysis. ZT and HL: manuscript writing. All authors read and approved the final manuscript.

## Conflict of Interest

The authors declare that the research was conducted in the absence of any commercial or financial relationships that could be construed as a potential conflict of interest.

## Publisher’s Note

All claims expressed in this article are solely those of the authors and do not necessarily represent those of their affiliated organizations, or those of the publisher, the editors and the reviewers. Any product that may be evaluated in this article, or claim that may be made by its manufacturer, is not guaranteed or endorsed by the publisher.

## Footnotes

1 http://bioconductor.org/packages/release/bioc/html/DESeq2.html

2 http://www.bioconductor.org/packages/release/bioc/html/goseq.html

3 http://kobas.cbi.pku.edu.cn/download.php

4 http://www.cytoscape.org/download.html

5 https://gephi.org/users/download/

6 https://en.freedownloadmanager.org/Windows-PC/OLIGO.html

## Supplementary Material

Supplementary Figure 1 GO and KEGG pathway enrichment analysis of tissue-specific genes. **(A)** GO enrichment analysis of tissue-specific genes (only the top 30 terms are shown based on corrected *P*-value, and terms with asterisks indicate significantly enriched terms). **(B)** KEGG pathway enrichment analysis of tissue-specific genes.

Supplementary Figure 2 Hierarchical clustering analysis of tissue-specific genes in different modules. **(A–G)** Hierarchical clustering analysis of bract-specific genes in the red module, leaf-specific genes in the brown module, petal-specific genes in the green module, pistil-specific genes in the salmon module, sepal-specific genes in the midnight blue module, stamen-specific genes in the turquoise module, and shoot-apex-specific genes in the yellow module, respectively.

Supplementary Figure 3 KEGG pathway enrichment analysis of tissue-specific genes in different co-expression modules (only significantly enriched pathways are shown). **(A–G)** KEGG pathway enrichment analysis of bract-specific genes in the red module, petal-specific genes in the green module, sepal-specific genes in the midnight blue module, shoot-apex-specific genes in the yellow module, leaf-specific genes in the brown module, pistil-specific genes in the salmon module, and stamen-specific genes in the turquoise module.

Supplementary Figure 4 Co-expression networks of tissue-specific genes in specific modules. Yellow, green, salmon, brown, red, turquoise, and midnight blue dots represent hub genes in the yellow, green, salmon, brown, red, turquoise, and midnight blue modules, respectively.

Supplementary Table 1 Primers used for RT-qPCR validation.

Supplementary Table 2 Information about the PacBio sequencing data.

Supplementary Table 3 Transcript lengths before and after correction by Illumina reads.

Supplementary Table 4 Statistics of the GMAP mapping results.

Supplementary Table 5 Information of twenty hub genes in specific co-expression modules.
