# Supplementary figures and images for: A Tissue-Specific Landscape of Alternative Polyadenylation, lncRNAs, TFs, and Gene Co-expression Networks in Liriodendron chinense
Source: Front Plant Sci. 2021 Jul 23;12:705321. doi: 10.3389/fpls.2021.705321 (PMC8343429; doi:10.3389/fpls.2021.705321)

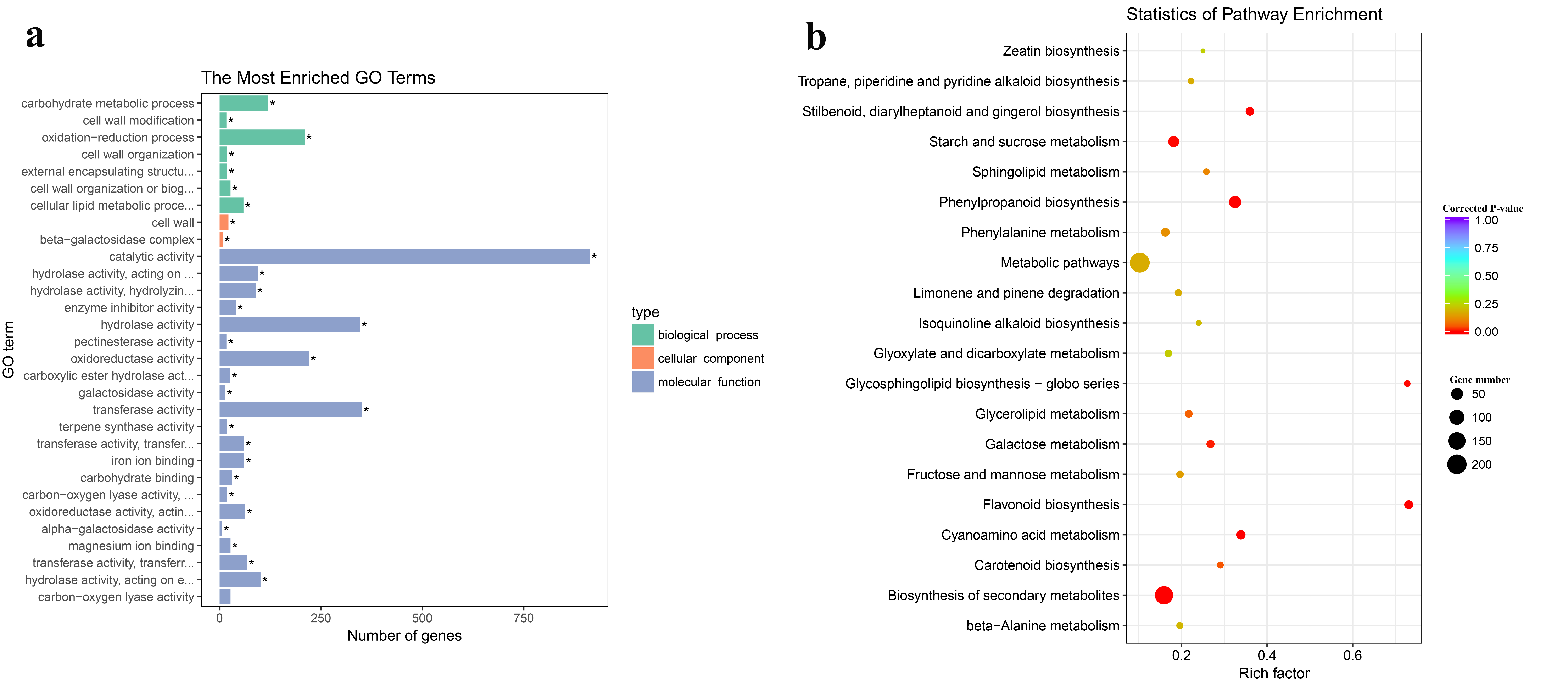

Supplement: Supplementary Figure 1 — GO and KEGG pathway enrichment analysis of tissue-specific genes. (A) GO enrichment analysis of tissue-specific genes (only the top 30 terms are shown based on corrected P-value, and terms with asterisks indicate significantly enriched terms). (B) KEGG pathway enrichment analysis of tissue-specific genes. [file Image_1.JPEG]

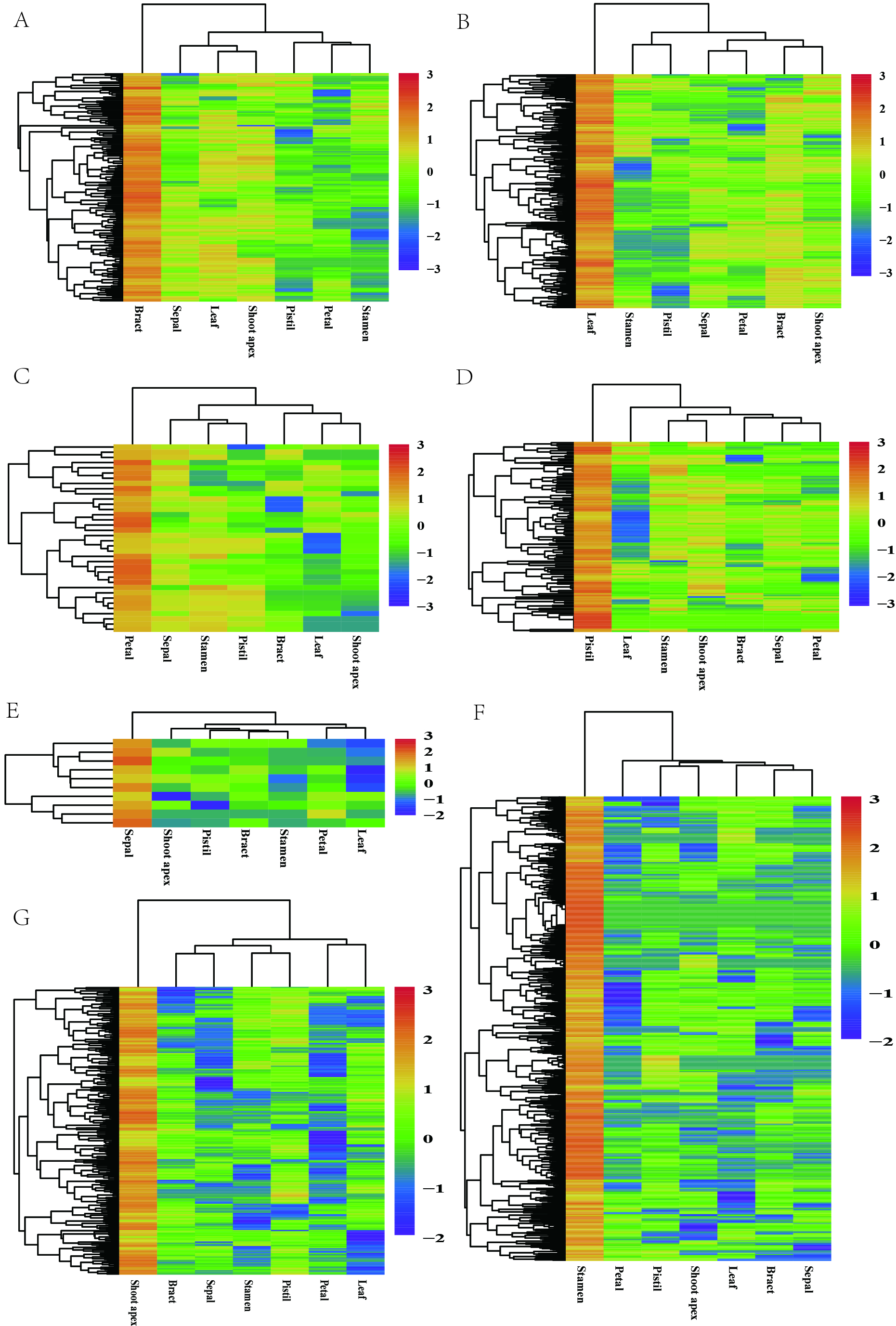

Supplement: Supplementary Figure 2 — Hierarchical clustering analysis of tissue-specific genes in different modules. (A–G) Hierarchical clustering analysis of bract-specific genes in the red module, leaf-specific genes in the brown module, petal-specific genes in the green module, pistil-specific genes in the salmon module, sepal-specific genes in the midnight blue module, stamen-specific genes in the turquoise module, and shoot-apex-specific genes in the yellow module, respectively. [file Image_2.JPEG]

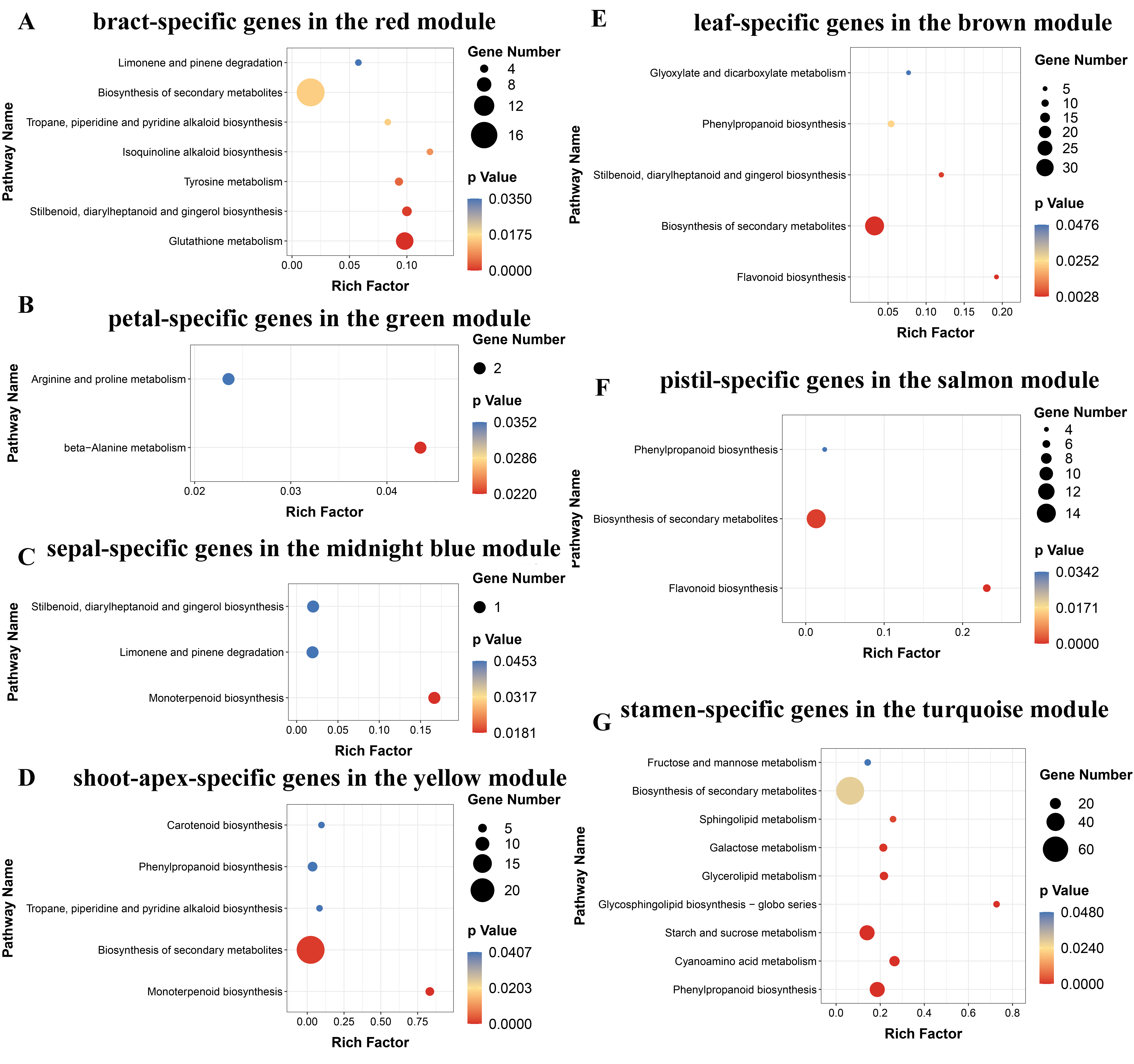

Supplement: Supplementary Figure 3 — KEGG pathway enrichment analysis of tissue-specific genes in different co-expression modules (only significantly enriched pathways are shown). (A–G) KEGG pathway enrichment analysis of bract-specific genes in the red module, petal-specific genes in the green module, sepal-specific genes in the midnight blue module, shoot-apex-specific genes in the yellow module, leaf-specific genes in the brown module, pistil-specific genes in the salmon module, and stamen-specific genes in the turquoise module. [file Image_3.JPEG]
