# Supplementary material for: A Tissue-Specific Landscape of Alternative Polyadenylation, lncRNAs, TFs, and Gene Co-expression Networks in Liriodendron chinense
Source: Front Plant Sci. 2021 Jul 23;12:705321. doi: 10.3389/fpls.2021.705321 (PMC8343429; doi:10.3389/fpls.2021.705321)
Supplement: Supplementary Table 1 — Primers used for RT-qPCR validation. [file Table_1.DOC]

**Table S1** Primers used for RT-qPCR validation.

| Gene | Primer sequence (5´ – 3´) |
| --- | --- |
| Lchi19253 | F: CGACGCTGGTGAGATTGTTTCC |
|  | R: CTTGGGTGTGTGCGATTTGTGC |
| Lchi22861 | F: TGGCAGTTCGGGCAGACCTT |
|  | R: TCCTCATTCAAACGCTCCCTCCT |
| Lchi16788 | F: CCCAATCTTTCCCGCTCTTTCTC |
|  | R: AGCCTCCTCCGCATCCTTCT |
| Lchi03883 | F: GCATCATACGCTCCACGAATCA |
|  | R: CGCCTAATCCGCACCCAACT |
| Lchi15751 | F: AGCAAGAGCCGCAAGAAGAGTATT |
|  | R: AGGAGATTAGAGACCAAGTGAGACCA |
| Lchi28227 | F: AAGCCTGTGAGTAAGCGGTGAT |
|  | R: AGGAGATTAGAGACCAAGTGAGACCA |
| Lchi28024 | F: CTGTCTGTCTCTGTCTCTGTCTCC |
|  | R: TGAACTGAAGCACAAACCCAATGA |
| Lchi03200 | F: AGTGAAGGAGGGAAGCAAGGTG |
|  | R: AGCGGTCTGGAAGCATCATAGC |
| Lchi20665 | F: GCAGAGCAGCAGAAGGTTACAGT |
|  | R: TCGTCCTCATCGTCAAAGTCATCC |
| Lchi02497 | F: CTACAAGAGAACAGGCAAGCA |
|  | R: CTTCTCCCCTTCCTCTACACC |
| Lchi05696 | F: TTGTCTTCATCAACCTTGCTCT |
|  | R: ACTATTCAAAGCGAACGACCAC |
| Lchi07307 | F: TCCTCCACGCCCTCTACTACGA |
|  | R: TGCCCACCACTGCTCTTCTTCA |
| Lchi14077 | F: TCCCTTCCACCACCACCATTCC |
|  | R: GCAGCACCATACAGACCTCCCA |
| Lchi21626 | F: TGCCCTTTTGCTGATGAAATGTCT |
|  | R: ATTGAAGTGAAGAAAGCCTTGGT |
| Lchi20614 | F: CCTCATCCCCTTCCCTCGTCT |
|  | R: CGCACACGCTCGACTTCACA |
| Lchi22115 | F: ATCATCTCAACCCCTATCCCT |
|  | R: GATTTTCCTTTCCGGCGTCT |
| eIF3 | F: CATCCAATTTCACTTTCCGCTCCAC |
|  | R: AATCACCAGCAGACGAGAAGCA |
