# Supplementary material for: A Tissue-Specific Landscape of Alternative Polyadenylation, lncRNAs, TFs, and Gene Co-expression Networks in Liriodendron chinense
Source: Front Plant Sci. 2021 Jul 23;12:705321. doi: 10.3389/fpls.2021.705321 (PMC8343429; doi:10.3389/fpls.2021.705321)
Supplement: Supplementary Table 2 — Information about the PacBio sequencing data. [file Table_2.DOC]

**Table S2** Information about the PacBio sequencing data.

| Type | Number | Average length (bp) |
| --- | --- | --- |
| Polymerase reads | 618,876 | 38,373 |
| Subreads | 10,437,029 | 2,177 |
| CCSs | 498,059 | 2,755 |
| FLNC reads | 430,554 | 2,568 |
| Polished consensus reads | 227,276 | 2,697 |
