# Supplementary material for: A Tissue-Specific Landscape of Alternative Polyadenylation, lncRNAs, TFs, and Gene Co-expression Networks in Liriodendron chinense
Source: Front Plant Sci. 2021 Jul 23;12:705321. doi: 10.3389/fpls.2021.705321 (PMC8343429; doi:10.3389/fpls.2021.705321)
Supplement: Supplementary Table 3 — Transcript lengths before and after correction by Illumina reads. [file Table_3.DOC]

**Table S3** Transcript lengths before and after correction by Illumina reads.

| Type | Total number | Mean length (bp) | Min length (bp) | Max length (bp) | N50 (bp) |
| --- | --- | --- | --- | --- | --- |
| Before correction | 227,276 | 2,697 | 180 | 14,478 | 3,107 |
| After correction | 227,276 | 2,696 | 180 | 14,735 | 3,105 |
