# Supplementary material for: A Tissue-Specific Landscape of Alternative Polyadenylation, lncRNAs, TFs, and Gene Co-expression Networks in Liriodendron chinense
Source: Front Plant Sci. 2021 Jul 23;12:705321. doi: 10.3389/fpls.2021.705321 (PMC8343429; doi:10.3389/fpls.2021.705321)
Supplement: Supplementary Table 4 — Statistics of the GMAP mapping results. [file Table_4.DOC]

**Table S**4 Statistics of the GMAP mapping results.

| Total reads | Total mapped reads | Unmapped reads | Multiply mapped reads | Uniquely mapped reads | Reads mapped to ‘+’ | Reads mapped to ‘-’ | Reads mapped  to known genes | Reads mapped to novel genes |
| --- | --- | --- | --- | --- | --- | --- | --- | --- |
| 227276 | 224,704 (98.87%) | 2,572 (1.13%) | 22,628 (9.96%) | 202,076 (88.91%) | 119,840 (52.73%) | 82,236 (36.18%) | 144,006  (63.36%) | 31,792  (13.99%) |
