# Supplementary material for: A Tissue-Specific Landscape of Alternative Polyadenylation, lncRNAs, TFs, and Gene Co-expression Networks in Liriodendron chinense
Source: Front Plant Sci. 2021 Jul 23;12:705321. doi: 10.3389/fpls.2021.705321 (PMC8343429; doi:10.3389/fpls.2021.705321)
Supplement: Supplementary Table 5 — Information of twenty hub genes in specific co-expression modules. [file Table_5.DOC]

**Table S5** Information of twenty hub genes in specific co-expression modules

| Gene | Specificity | Annotation |
| --- | --- | --- |
| *Lchi00262* | Bract | Pathogenesis-related protein |
| *Lchi05264* | Bract | Leucine-rich repeat receptor-like protein kinase |
| *Lchi04173* | Bract | L-type lectin receptor-like kinase |
| *Lchi12717* | Bract | ECERIFERUM 1-like protein |
| *Lchi02151* | Bract | Purine permease 1 |
| *Lchi00205* | Leaf | Unknown |
| *Lchi03539* | Leaf | Nudix hydrolase |
| *Lchi03373* | Leaf | Glyceraldehyde-3-phosphate dehydrogenase |
| *Lchi00122* | Leaf | Chloroplast stem-loop binding protein |
| *Lchi05497* | Leaf | Ribulose bisphosphate carboxylase/oxygenase |
| *Lchi02846* | Leaf | Wall-associated receptor kinase-like |
| *Lchi11008* | Petal | Short-chain dehydrogenase/reductase SDR |
| *Lchi02536* | Pistil | Protein LAZY 1 |
| *Lchi17170* | Sepal | Alpha-terpineol synthase |
| *Lchi05072* | Shoot apex | AINTEGUMENTA-LIKE 5 |
| *Lchi02885* | Shoot apex | Expansin A1 |
| *Lchi00871* | Shoot apex | Cinnamoyl-CoA reductase 1 |
| *Lchi00754* | Shoot apex | Unknown |
| *Lchi00698* | Shoot apex | LRR receptor-like serine/threonine-protein kinase |
| *Lchi25777* | Stamen | SPOROCYTELESS/NOZZLE |
